# Tumor of Inferior Maxillary

**Published:** 1872-08

**Authors:** 


### EDITORIAL DEPARTMENT.

---

*Tumor of Inferior Maxillary.*—Dr. T. H. Parramore sends the following account of a case of this kind which recently came under his observation:

"I was called upon by a physician a few days ago to examine the mouth of a young lady (about fifteen) who has a tumor on the right side of her face covering the lower maxilla from the first molar tooth to the angle of the jaw; this tumor first made its appearance about three or four months ago; it is very hard externally and although not so large, it can be felt on the inside of the jaw, by passing the finger along the lingual side of the inferior maxilla to the root of the second molar tooth, which is quite loose and has its crown thrown inwards, indicating that its roots have been raised in their sockets and pushed outwards. I could detect a slight crepitation when moving this tooth; the first molar, although tolerably firm, in color would indicate a loss of vitality; her gums are apparently in a healthy condition; her breath is quite fetid, there is no escape of pus, and it has never given any pain except when the loose tooth is moved, which she says causes this tumor to pain her slightly. I cannot discover any indications of scrofulous or any other constitutional vice, and the physician above referred to tells me that her parents have no constitutional derangement whatever. He (the physician) prescribed wine of iron internally and camphoreted mercurial ointment externally. From the symptoms I would consider it a case of osteo-sarcoma."

[The remedy for epulis and tumors of like nature is early and thorough excision of the portion of the bone in which they have its origin.—Ed.]

